# Prof. Schenck’s Researches on the Predetermination of Sex

**Published:** 1898-09

**Authors:** 


### PROF. SCHENCK'S RESEARCHES ON THE PREDETERMINATION OF SEX.

Prof. S. treats the subject under three heads, a summary of the writings of his predecessors, an account of his own researches and deductions, and thirdly, a description of the method of treatment he has devised, with cases.

In the first part, he agrees with the conclusions of various writers that, if the sexual power of the male be greater a female offspring is more likely to result, and *vice versa*. With regard to environment upon sex, in warm climates females predominate, in cold and unfavorable males. The second part begins with the enunciation of the fact observed in domestic animals and in insects, that the better the mother is nourished the more females she produces, the number of males remaining constant. Schenck set out upon a series of observation based on the theory of crossed sexual inheritance. He first investigated the excreta and particularly the carbohydrates of the urine. The presence of a certain amount of sugar, which is commonly recognizable by the phenylhydrazine test in perfectly normal individuals, indicates incompleteness of the oxidation process whereby a certain quanity of heat is lost to the body. Now the quality of sugar normally excreted is equal in men and women, but more significant in the latter owing to the lesser activity of their metabolic processes. For the perfect ripening of the ovum, it is necessary that oxidation shall be perfect; that is, that no sugar shall be left unburnt. Where there is a remainder of unburnt sugar the ovum stands a chance of being less ripe and well nourished. Hence the properties of its protoplasm are less well developed, and by the theory of crossed inheritance it is more likely to produce a female child. On the other hand, when the urine is free from sugar the ovum can attain perfect development and give rise to male offspring. It is upon this cardinal principle that Schenck's theory is based. He holds that a prolonged course of appropriate nourishment both before and after fertilization will tend to the conception of male children only. The next question is of the means to be adopted. If a male child is desired and the maternal urine contains no sugar but abundance of reducing substances (particularly the levorotatory glycuronic acid) he allows impregnation forthwith. If, on the other hand, sugar is present, it must be removed. Finally, Schenck gives his clinical results. He quotes numerous cases to show that the bearing of female children is associated with glycosuria. In such cases, he recommends a diet comprising plenty of proteid and fat and as little carbohydrate as can be tolerated. This must be taken for two or three months before and after impregnation. He concludes after giving examples (such as, in one family where six boys had been born; under this theory and treatment a girl immediately followed). In countries where much flesh is consumed there is a marked prepondance of male children. The birth of male children can thus in certain cases be predetermined, but the voluntary production of girls is a problem as yet unsolved.—*Medical Review.*

---

Jonathan Hutchison, in his *Archives of Surgery*, says that he has long been in the habit of prescribing coffee as a medicine in certain states of great debility. He regards it as a remedy quite unique in its usefulness in sustaining the nervous energy in certain cases. Apart from its general utility, and its well-known value as an antidote to opium, he has found it of especial service after operations where anesthetics had been used, and in states of exhaustion where alcohol had been pushed and a condition of semi-coma followed. In these latter cases he has sometimes prescribed it as an enema when the patient could not swallow, and with the best effects. In many cases where death may be close at hand, such an expedient as this may even be the means of permanent restoration to health. Tea and coffee seem to be much alike, in many respects, but the latter is greatly preferable as to its sustaining power. It would be a great advantage to our working classes, and a great help toward the further development of social sobriety, if coffee were to come into greatly increased use, and if the ability to make it well could be acquired. As an example of the difference of effect of tea and coffee upon the nerves, the writer notes what he believes many sportsmen will confirm, that it is far better to drink coffee than tea when shooting. Tea, if strong, or in any quantity, especially if the individual be not in very robust health, will induce a sort of nervousness which is very prejudicial to steady shooting. Under its influence one is apt to shoot too quickly, whereas coffee steadies then had and gives quiet nerves.—*Medical Times.*

